# Influence of Education on Dental Anxiety and Fear in Mental Disorders after Viewing a Molar Extraction in Three Types of Mental Disorder Compared to Persons with No Mental Disorder

**DOI:** 10.3390/jcm13133868

**Published:** 2024-06-30

**Authors:** Elena Bermúdez-Bejarano, Juan-Antonio Bermúdez-Sánchez, Francisco-José Ruiz-Rey, María Baus-Domínguez, María-Ángeles Serrera-Figallo, José-Luis Gutiérrez-Pérez, Daniel Torres-Lagares

**Affiliations:** 1 Stomatology Department, Faculty of Dentistry, Sevilla University, C/Avicena s/n, 41009 Seville, Spain; elenabermudezbejarano@hotmail.com (E.B.-B.); mbaus@us.es (M.B.-D.); jlgp@us.es (J.-L.G.-P.); danieltl@us.es (D.T.-L.); 2 Private Practice in Psychiatry, C/Peinado, 42 2ªF, 29014 Málaga, Spain; jabsmeba@hotmail.com; 3 Department of Didactics of Mathematics, Didactics of Social Sciences and Experimental Sciences, Avda. Cervantes, 2, 29071 Málaga, Spain; pacoruizster@gmail.com; 4 Oral and Maxillofacial Unit, Virgen del Rocio Hospital, 41013 Seville, Spain

**Keywords:** dental anxiety, dental fear, education, anxiety level, adjustment disorder, mixed anxiety-depressive disorder, surgical extraction

## Abstract

**Objective:** The goal of this study is to validate the psychometric properties of the Modified Dental Anxiety Scale (MDAS) and the Dental Fear Scale (DFS) at three different times: seven days before, immediately after, and seven days after watching a video of surgical extraction of a lower third molar in a sample with four strata: anxiety disorder, adjustment disorder, mixed anxiety-depressive disorder, and no mental disorder ages 18–70 in a psychiatric clinic in Malaga. **Methods:** The Research Ethics Committee of the Virgen Macarena-Virgen del Rocío Hospitals approved the study. After being interviewed by a psychiatrist and subsequently completing the Hamilton Rating Scales for Anxiety and Depression, the participants were divided into 4 strata (60 persons in each). The influence of education level was then analyzed. **Results:** The scales demonstrated good psychometric properties, with higher MDAS and DFS scores for lower levels of education and mixed anxiety-depressive disorder. **Conclusions:** Patients who show higher levels of dental anxiety and dental fear will be those with lower education levels, as well as those who suffer from mixed anxiety-depressive disorder.

## 1. Introduction

Dental anxiety and dental fear are two constants in dental clinics, and they are more common than we think. When they appear, they are sometimes associated with generalized anxiety disorder. When high general anxiety is present, dental anxiety and dental fear will be present as well [1], translating into a decrease in the quality of preexisting oral health and, thus, of the patient’s quality of life [2,3,4]. These anxieties and fears will also depend on the type of procedure to be performed and are more likely to appear in invasive therapies such as oral surgery than in less invasive treatments. Within oral surgery, dental extraction is perceived as one of the five dental procedures that awaken the most fear of having a stressful experience due to its physical and psychological impact [5,6]. Extraction causes more dental anxiety than drilling a tooth or injecting local anesthetic [6], as it is a very upsetting surgical procedure with potential risks and complications that can affect the patient [7]. Among extractions, extraction of the lower third molar causes the most anxiety [8], and it is a widespread procedure that involves a series of surgical challenges (depth of impaction, distal space, and angulation of the third molar, among others) [9].

When expressed, dental anxiety and dental fear are usually an obstacle between dentist and patient, as dental anxiety can trigger a stress reaction that can, in turn, generate anxiety, fear, and other factors, such as coping mechanisms, loss of self-control, and unpleasant emotions (feeling unhappy, insecure, guilty, etc.) [10]. All of these factors influence reduction, deferral, avoidance, and postponement of appointments, contributing to a vicious cycle that worsens oral health and may contribute to a decline in mental health [2,3,10,11,12,13,14], with a clear association between dental anxiety, general anxiety and fears, neuroticism, and general psychological anxiety [15]. If these problems occur in a patient free of mental disorders, we can expect them to be much worse in patients with a mental disorder and possibly even to increase the disorder.

Some studies confirm that individuals who watch videos providing pre-operatory information that includes the steps of the procedure to be performed can experience anxiety before viewing the video [16,17,18,19,20]. Other studies argue that dental anxiety may be more significant after the surgery due to the type and duration of the procedure performed, the anesthetic, and the position of the tooth extracted [14]. If this anxiety occurs in a patient with no mental disorder, what might happen in one who suffers from a disorder? According to the 2016 study by Kyle et al., depression, dental fear, and the state of anxiety have a marked influence on dental pain [21], as does being a patient with high sensitivity to dental anxiety [22]. Attention must thus be paid to these issues.

This study combines an analysis of three types of mental disorders: anxiety disorder, adjustment disorder, and mixed anxiety-depressive disorder. In anxiety disorder, anxiety appears in the presence of any eventuality that threatens identity and/or aggression against the self. If anxiety becomes intense, frequent, or persistent and interferes with daily life, it can become part of an anxiety disorder. Adjustment disorder, in contrast, is a phenomenon always related to stress. It causes mental maladjustment and symptomology that remain until the stressor disappears or the person adjusts anew. The clinic usually inspires a depressive mood, anxiety, worry, and a feeling of inability to cope, plan the future, or continue in the current situation. This feeling was previously termed secondary depression or endogenous depression, whereas mixed (anxiety-depressive) disorders were previously termed endogenous depression and indicate disorders that develop symptoms of both disorders without the apparent predominance of either [23].

It is thus essential to diagnose dental anxiety and dental fear as soon as possible to avoid the vicious cycle mentioned above. Mental disorders must also be diagnosed to determine their evolution and establish therapy promptly. Hetero-administered scales exist to diagnose a mental disorder, can be completed by patients themselves, and have good psychometric properties. The Hamilton Rating Scale for Anxiety (HRS-A) and the Hamilton Rating Scale for Depression (HRS-D) are two such scales. Both were validated in Spanish by Lobo et al. in 2002 and by Ramos Brieva et al. in 1988 correlatively [24,25]. The current study’s authors participated in a recent study to validate these scales. They obtained optimal results that showed high reliability and construct validity for the three video viewing times [26]. To evaluate the level of dental anxiety and dental fear, we used the Modified Dental Anxiety Scale (MDAS) and Dental Fear Scale (DFS) scales, respectively. These scales were also validated in Spanish and obtained good psychometric properties [27].

This study aims to add valuable statistical data and continue the study that we published in August 2022. The current study will evaluate the sociodemographic influence of education level and levels of dental anxiety and dental fear using the MDAS and DFS in the four sample groups chosen (adjustment disorder, anxiety disorder, anxiety-depressive disorder, and no mental disorder) after watching a video of a surgical extraction of a lower third molar at three very different times (seven days before viewing, after viewing, and seven days after viewing), as well as the clinical implications of this influence. It will also examine the psychometric properties of the MDAS and the DFS.

## 2. Materials and Methods

### 2.1. Sample Selection and Protocol Followed

This prospective observational study was performed at a private psychiatric clinic in Malaga. It ran from October 2019 to January 2020. The study identified 240 Caucasians ages 18–70 and invited them to participate, promising complete anonymity and informed consent.

The sample was divided into 60 participants per group, according to the preliminary interview performed by the psychiatrist (J.A.B.S.) and the patient’s completion of the HRS-A or HRS-D scales to avoid bias on the part of the psychiatrist. The participants were classified into adjustment disorder, anxiety disorder, anxiety-depressive disorder, and no disorder. This sample was chosen to facilitate the statistical analysis based on all study variables.

Participants completed the scales mentioned above and the MDAS and DFS one week before, immediately after, and one week after watching the video.

The goal was to evaluate any possible change in the evolution of mental disorders, as well as the patients’ levels of dental anxiety and fear, and to determine whether education influenced the result.

The video showed the surgical extraction of a partially impacted lower third molar. It lasted 2 min and 21 s and contained information about the procedure to be performed, from the scalpel incision to ostectomy, dental sectioning, and finally, suture. The video belongs to the three authors of this article, who are oral surgeons (E.B.B., D.T.L., and J.L.G.P.), and can be viewed at this permalink: https://youtu.be/YriQxJwUPoY (accessed on 31 March 2022).

### 2.2. Ethics

This study follows the Declaration of Helsinki: Declaration of Helsinki: Ethical principles for medical research involving human subjects. The protocol was approved by the Research Ethics Committee of the Virgen Macarena-Virgen del Rocío Hospitals on 14 January 2019, with approval code 76030446e8bf49e55f4b0ecab4b6fc43b4128ffd.

### 2.3. Statistical Analysis

The statistical analysis used SPSS 21.0 software (IBM, Armonk, New York, NY, USA).

The ANOVA test was used to evaluate the sociodemographic factor (education), as the study involved three or more independent samples. Thus, the following descriptive tables were designed for Tukey’s HSD post hoc Test.

We assume the means significantly differ when the significance is less than 0.05 in the different items. This test analyzes education levels, classified as Group 1 (primary education/no education), Group 2 (compulsory and noncompulsory secondary and vocational education), and Group 3 (tertiary education or university study).

The article we published in August 2022 in the Journal of Clinical and Experimental Dentistry analyzed the HRS-A and HRS-D, obtaining good psychometric properties with outstanding reliability, construct validity, and factor extraction. For the three video viewing times, the MDAS and DFS both obtained a reliability score of above 0.85, indicating optimal Alpha Cronbach values. This confirms the items as valuable tools for measuring what they seek to measure and thus demonstrates the scales’ good internal consistency.

We used the KMO Index for construct validity, which should be above 0.5 to continue with factor analysis. Both scales yielded values well above this index. Extraction factors aim to explain the maximum total variance by identifying a low number of components. The Varimax method and a rotated components matrix were used to determine which variables might be included or discarded. Values should be above 0.5 to retain the items.

Along with these statistical data, we gathered more data using the HRS-A, HRS-D, MDAS, and DFS for the variables age and medication readjustment, on which we hope to publish future articles.

## 3. Results

### 3.1. Sociodemographic Factor

Appendix A (Table A1, Table A2, Table A3, Table A4, Table A5, Table A6 and Table A7) contains the statistical results obtained and the corresponding tables of means and standard deviations.

#### 3.1.1. Results of the Hamilton Rating Scale for Anxiety (HRS-A) and the Hamilton Rating Scale for Depression (HRS-D)

The results for the influence of education level on response to the video at the three viewing times using the HRS-A were as follows. Seven days before watching the video, Group 1 obtained higher means than Groups 2 and 3 on all items. After watching the video, Group 1 had higher means for all items than Groups 2 and 3, except Item 1 (anxious), where Group 2 had the highest values. Seven days after watching the video, Group 1 had higher means for all items than Groups 2 and 3, except for Items 9 (cardiovascular symptoms), 10 (respiratory symptoms), and 14 (behavior during the interview), for which Group 2 obtained the highest values. Group 1 (primary education/no education) thus had a higher level of anxiety measured by the HRS-A than Group 2 (compulsory and noncompulsory secondary and vocational education) and Group 3 (university study).

#### 3.1.2. Results of the Hamilton Rating Scale for Depression (HRS-D)

The HRS-D results on education level relevance for the three screening times were as follows. Seven days before watching the video, Group 1 (primary education/no education) had higher means on all items than Group 2 (compulsory and noncompulsory secondary and vocational education) and Group 3 (tertiary/university study), except on Item 12 (gastrointestinal somatic symptoms), for which Group 2 obtained the highest mean. After watching the video, Group 1 had higher means for all items than Groups 2 and 3 except Items 9 (agitation), 10 (psychic anxiety), 11 (somatic anxiety), and 13 (general symptoms), for which Group 2 had higher means. Seven days after watching the video, Group 1 had higher values on all items than Groups 2 and 3. The HRS-D thus measures a higher level of depression in Group 1 (primary education/no education) than in Group 2 (secondary education) or Group 3 (university study).

These results on the means and standard deviations of the HRS-A and HRS-D for responses to watching the video three times are shown in Table A1, Table A2 and Table A3 and correspond to the means and standard deviations of the HRS-A and HRS-D scales obtained for the three viewing times.

#### 3.1.3. Results of the Modified Dental Anxiety Scale

The results for the effect of education level, obtained using the MDAS scale at the three video viewing times, were as follows: Seven days before watching the video, Group 1 (primary education/no education) had higher means for all items than Group 2 (secondary education) and Group 3 (higher/university education). Group 1 also had the highest values at the other two viewing times, immediately after watching the video and seven days later. Group 1 (primary education) thus has a higher level of dental anxiety than Group 2 (secondary education) and Group 3 (higher/university education).

#### 3.1.4. Results of the Dental Fear Scale

The results for the DFS showed the following impact of education level at the three video viewing times. Seven days before viewing, Group 1 (primary education) obtained higher means for all items than Group 2 (secondary education) and Group 3 (higher/university education). After viewing the video, Group 1 (primary education) also had the highest means, except for Items 5 (I perspire… I sweat), 20 (considering everything, fear of dental treatment), and 24 (your siblings’ fear), for which Group 2 (secondary education) obtained higher means. Seven days after watching the video, Group 1 (primary education/no education) had the highest means on all items except Item 24 (your siblings’ fear), for which Group 2 (secondary education) obtained the highest. The highest level of dental fear thus occurs in Group 1 (primary education/no education). This level is higher than in Group 2 (secondary education) or Group 3 (higher/university education).

The results are displayed in Table A4, Table A5 and Table A6, representing the means and standard deviations obtained using the MDAS and DFS for the three video viewing times.

### 3.2. Psychometric Properties

The MDAS values were grouped into a single factor for all three viewing times and were unidimensional with an accumulated variance of 80–88%. The DFS was grouped into two factors with an accumulated variance of 71–79%, except for the middle viewing time (just after the video screening), where we propose eliminating two items, as will be explained in the discussion. We need not eliminate any other item from the two scales used. The results of the psychometric properties are displayed in Table A7.

## 4. Discussion

### 4.1. Education Level

Our findings agree with the literature reviewed in finding that higher education level correlates with less dental anxiety and fear and will thus have less influence on a person’s general psychological state and/or mental disorder(s). This effect will translate into a more psychologically prepared patient for relevant dental treatment. Because some authors argue, however, that patients with primary education have a lower level of dental anxiety and fear, we will now attempt to differentiate these two positions.

First, we note that the negative correlation some studies find between the connection between the level of dental anxiety and education level is that people with a higher education level (more education) have a higher level of anxiety [8,28,29,30,31,32,33,34,35]. This result may be due to other reasons, such as fear of a cross infection [36]. Other studies do not find a clear association between dental anxiety and education level [37,38,39] and argue that the association is due to different causes, such as the calculation index [39], which relates the level of dental anxiety to the periodontal condition [40].

Second, some studies with a positive correlation argue that people with higher education levels are more aware of medical and dental services [41] and will thus be more likely to attend dental appointments regularly [41,42]. Giving individuals better tools for facing stressful situations such as dental anxiety and fear and decreasing their levels of these feelings [43] results in more care for the individual’s oral health, such that dental anxiety and fear will be inversely proportional in individuals with a lower education level [6,43,44,45,46,47,48,49,50,51,52]. We thus find the greater prevalence of dental phobia in patients with low education levels [53] and consider the possibility that education acts as a protection factor against fear and anxiety [14]. Other authors, however, deny a statistically significant relationship between education level and dental fear [30,54,55] or dental anxiety based on completion of the MDAS [49].

Further, some studies analyze the association between the type of job and level of dental anxiety, revealing that retired subjects, homemakers, and unemployed subjects differ from individuals who are working or have work outside the home; the first group has higher dental anxiety [51,56]. This relationship has also been identified for dental fear, as some research observes that unemployed individuals have a higher level of dental fear [50]. Similar findings have been observed in patients diagnosed with depression or anxiety, where the unemployed, the retired, those unable to work, homemakers, and students are more likely to suffer from this type of disorder than people who work outside the home [57]. Research thus finds an association between dental anxiety and fear, general anxiety and fear, and general psychological state (depression, anxious state, anxious trait, etc.) [46,52,58].

Thus, although controversy exists in the literature reviewed, most studies to date on the issue confirm that education level is an essential factor in evaluating dental fear and anxiety [49]. Patients with primary education only usually have a higher level of dental anxiety [59] and higher scores on the Dental Anxiety Scale (DSA) and the DFS than patients with secondary education (university and postgraduate) and patients with lower socioeconomic status. This finding may occur because these patients have less dental awareness, causing increased dental anxiety and fear during dental treatments [43,49,52] and irregular attendance of dental appointments [60].

We have also observed that patients who have anxiety-depressive disorder are more inclined to develop a higher level of dental fear than patients who have only anxiety disorder, only depressive disorder, or do not suffer from these disorders [46,52]. Dental anxiety thus correlates with depressive disorders [61] and anxiety disorders [62].

### 4.2. Psychometric Properties

The statistical values for the reliability of the MDAS at the three video viewing times are 0.939, 0.954, and 0.967, respectively. We find high internal consistency and an Alpha Cronbach well above the coefficients recorded in the literature, which range from 0.79 to 0.88 for the Portuguese [34], Spanish [63], and Turkish [49] translations. The values for the DFS are 0.984, 0.890, and 0.926—values very similar to those in the literature, where they range from 0.95 to 0.97 in the Turkish [30], Greek [64], Japanese [65], and Norwegian [66] translations.

For construct validity of the MDAS for the three viewing times, our statistical results are 0.962, 0.868, and 0.870, respectively. The values for the DFS are 0.841, 0.964, and 0.957. Because the Spanish version of the scale [27] uses Spearman’s test, we could not compare it. The 2009 study by Humphris et al. obtained a KMO Index of 0.842 for the MDAS and the DFS and again used Spearman’s text [38]. Given the different statistical instruments used, we could not compare our results to those of these articles.

Our results for factor extraction for the MDAS for the three viewing times show that the confirmatory analysis of the five items can be grouped into a single factor. We thus need not eliminate any item, as the coefficient is greater than or equal to 0.5, with an accumulated variance of 80.5–88.406%. This result agrees with the articles consulted and defines the items’ strong unidimensional character [67]. For the DFS, we propose that the confirmatory analysis can be grouped into two factors with accumulated variance ranging from 71.523 to 79.281%. These results disagree with their 1978 creator, Kleinknecht et al., who grouped the data into three factors [68], as did Milgrom et al. in 1990 [69]. We propose eliminating two items in the middle of the time studied (immediately after the video screening): Item 20 (On considering all of these things, what are you afraid of in having dental treatment?) and Item 24 (your siblings’ fear), since the values for this dimension are 0.141 and 0.139, respectively. We thus do not eliminate any factor for the times seven days before viewing the video and seven days after it. We cannot compare these results to the literature reviewed.

The marked controversy in the literature regarding the statistical analysis performed may be due to the great variety of measurement instruments used, the selection of the samples, and possible mental disorders of the individuals in them.

### 4.3. Limitations of the Study

The present study has several limitations. First, the inclusion of only three types of mental disorders may not adequately represent the diversity of psychiatric conditions that can influence dental anxiety and fear. The use of self-administered questionnaires to measure anxiety and fear may be subject to response bias and lack of precision. Additional demographic factors may influence the study results. Socioeconomic status can significantly affect access to dental and dental and education health services. Patients with lower socioeconomic levels may have less access to regular dental care, increasing dental anxiety and fear, and may have fewer resources to access digital educational tools, limiting their potential benefit. Past dental experiences can also strongly influence current perception and anxiety; patients with previous traumatic experiences may have higher levels of fear and anxiety, regardless of their educational level. Age may affect patients’ ability to understand and benefit from digital educational interventions, as older patients may have less familiarity with digital technologies. Finally, cultural and ethnic differences may influence perceptions of dental health and access to education. Considering these additional factors may provide a deeper and more accurate understanding of the impact of education and other interventions on dental anxiety and fear in different populations.

However, including a control group without a mental health disorder can offer several potential benefits. A control group provides a baseline to compare dental anxiety and fear levels, helping to identify specific differences attributable to mental disorders. It allows one to evaluate the effectiveness of educational or visualization interventions in a population without mental disorders, providing valuable information on the general applicability of the interventions. It also helps identify specific factors that can exacerbate dental anxiety and fear in patients with mental disorders compared to those without such diagnoses. In addition, it allows for controlling additional confounding variables that could influence anxiety and fear levels, thus improving the validity of the results.

In the limitations of this study, it is important to note that numerous statistical tests were performed to analyze the data. Although each test was performed with a conventional significance level set at <0.05, the large number of comparisons increases the possibility that some statistically significant results were due to chance. Therefore, caution is recommended when interpreting these findings and it is suggested that this possibility be considered when evaluating the differences observed in the various measures of dental anxiety and fear. This consideration is crucial to ensure a balanced and accurate understanding of the results presented.

## 5. Conclusions

The statistical results of our study confirm the good psychometric properties of the MDAS and the DFS, as well as the conclusion that patients with a higher education level (university study) experience lower levels of dental anxiety and dental fear than patients with a lower education level (primary education).

Similarly, a patient with a lower education level who also suffers from mixed (anxiety-depressive) disorder is more inclined to develop general anxiety and fear, dental anxiety and dental fear, and a more anxious psychological state than one who has only anxiety disorder, only depressive disorder, or no mental disorder.

## Data Availability

The data presented in this study are available upon request from the corresponding author (the data are not publicly available due to privacy and ethical restrictions).

## Appendix A

**Table A1:** Means and SDs for education level were obtained for the Hamilton scales for anxiety (Ham-Anx) and depression (Ham-Dep) seven days before viewing the video.

Scales/Items	Anxiety Disorder			Adjustment Disorder			Mixed Anxiety-Depressive Disorder			No Disorder		
HAM-ANX	F1	F2	F3	F1	F2	F3	F1	F2	F3	F1	F2	F3
Item 1	1.10 ± 0.316	1.04 ± 0.189	1.00 ± 0.000	1.00 ± 0.000	1.00 ± 0.000	1.00 ± 0.000	1.05 ± 0.218	1.10 ± 0.301	1.00 ± 0.000	1.00 ± 0.000	0.80 ± 0.523	0.06 ± 0.243
Item 2	1.10 ± 0.316	1.00 ± 0.272	0.73 ± 0.456	1.00 ± 0.000	0.95 ± 0.213	0.75 ± 0.452	1.10 ± 0.301	1.00 ± 0.000	1.06 ± 0.236	0.83 ± 0.388	0.35 ± 0.489	0.41 ± 0.507
Item 3	0.80 ± 1.033	0.36 ± 0.488	0.18 ± 0.395	0.62 ± 0.496	0.73 ± 0.456	0.33 ± 0.492	1.05 ± 0.384	1.10 ± 0.301	0.78 ± 0.647	0.52 ± 0.511	0.15 ± 0.366	0.00 ± 0.000
Item 4	1.40 ± 0.966	1.07 ± 0.262	1.09 ± 0.294	1.04 ± 0.344	1.14 ± 0.351	1.00 ± 0.000	1.95 ± 0.218	1.67 ± 0.483	1.39 ± 0.502	1.17 ± 0.388	0.85 ± 0.366	0.65 ± 0.493
Item 5	1.20 ± 0.789	1.07 ± 0.262	0.82 ± 0.395	0.77 ± 0.514	0.86 ± 0.560	0.92 ± 0.289	1.86 ± 0.359	1.48 ± 0.512	1.28 ± 0.461	1.00 ± 0.302	0.50 ± 0.513	0.18 ± 0.393
Item 6	0.80 ± 0.422	0.86 ± 0.356	0.41 ± 0.503	0.81 ± 0.402	0.73 ± 0.456	0.75 ± 0.452	1.43 ± 0.507	1.29 ± 0.463	0.89 ± 0.471	0.57 ± 0.507	0.00 ± 0.000	0.24 ± 0.437
Item 7	0.80 ± 0.422	0.64 ± 0.621	1.00 ± 0.000	0.85 ± 0.368	0.91 ± 0.294	0.83 ± 0.389	1.38 ± 0.498	1.14 ± 0.359	1.06 ± 0.236	0.96 ± 0.209	0.45 ± 0.510	0.59 ± 0.507
Item 8	0.40 ± 0.699	0.21 ± 0.418	0.23 ± 0.429	0.42 ± 0.504	0.23 ± 0.429	0.33 ± 0.492	1.19 ± 0.512	0.67 ± 0.577	0.56 ± 0.511	0.70 ± 0.470	0.25 ± 0.444	0.12 ± 0.332
Item 9	1.20 ± 0.422	1.11 ± 0.315	0.86 ± 0.351	1.00 ± 0.283	1.05 ± 0.213	1.00 ± 0.000	1.29 ± 0.561	1.19 ± 0.402	1.06 ± 0.416	0.96 ± 0.209	1.05 ± 0.224	0.76 ± 0.437
Item 10	1.70 ± 0.675	1.32 ± 0.476	1.05 ± 0.213	1.04 ± 1.96	1.00 ± 0.309	1.00 ± 0.000	1.00 ± 0.000	1.05 ± 0.218	1.00 ± 0.000	1.00 ± 0.000	0.95 ± 0.394	0.47 ± 0.514
Item 11	1.80 ± 0.919	1.25 ± 0.441	0.95 ± 0.213	0.92 ± 0.272	0.95 ± 0.375	0.83 ± 0.577	1.10 ± 0.301	1.10 ± 0.301	1.11 ± 0.323	0.74 ± 0.449	0.60 ± 0.503	0.06 ± 0.243
Item 12	0.80 ± 0.422	0.50 ± 0.509	0.50 ± 0.512	0.81 ± 0.491	0.36 ± 0.492	0.25 ± 0.452	1.19 ± 0.402	0.81 ± 0.512	0.89 ± 0.323	0.83 ± 0.388	0.25 ± 0.444	0.12 ± 0.332
Item 13	1.10 ± 0.316	1.07 ± 0.539	0.91 ± 0.426	0.92 ± 0.272	0.86 ± 0.351	0.50 ± 0.522	0.90 ± 0.539	0.76 ± 0.436	0.61 ± 0.502	0.39 ± 0.499	0.60 ± 0.503	0.06 ± 0.243
Item 14	1.00 ± 0.471	0.93 ± 0.378	0.68 ± 0.477	0.96 ± 0.196	1.05 ± 0.213	1.00 ± 0.000	1.43 ± 0.507	1.29 ± 0.463	1.11 ± 0.323	1.00 ± 0.000	1.05 ± 0.394	0.59 ± 0.507
**HAM-DEP**												
Item 1	0.90 ± 0.316	0.71 ± 0.460	0.55 ± 0.510	1.00 ± 0.000	0.86 ± 0.351	0.83 ± 0.389	1.00 ± 0.000	1.00 ± 0.000	1.00 ± 0.000	0.74 ± 0.449	0.20 ± 0.410	0.00 ± 0.000
Item 2	1.10 ± 0.568	0.61 ± 0.497	0.73 ± 0.456	0.92 ± 0.272	0.73 ± 0.456	0.67 ± 0.492	1.00 ± 0.000	0.95 ± 0.218	1.00 ± 0.000	0.17 ± 0.388	0.20 ± 0.410	0.12 ± 0.332
Item 3	0.40 ± 0.516	0.07 ± 0.262	0.09 ± 0.294	0.12 ± 0.326	0.05 ± 0.213	0.08 ± 0.289	0.29 ± 0.463	0.29 ± 0.463	0.28 ± 0.461	0.13 ± 0.344	0.05 ± 0.224	0.00 ± 0.000
Item 4	1.00 ± 0.000	0.96 ± 0.189	0.91 ± 0.294	1.00 ± 0.000	1.00 ± 0.000	1.00 ± 0.000	1.10 ± 0.301	1.14 ± 0.359	1.11 ± 0.323	1.00 ± 0.000	0.95 ± 0.224	0.41 ± 0.507
Item 5	1.00 ± 0.471	0.82 ± 0.390	0.45 ± 0.510	0.85 ± 0.368	0.82 ± 0.395	1.00 ± 0.000	1.10 ± 0.301	1.05 ± 0.384	1.06 ± 0.236	0.96 ± 0.209	0.35 ± 0.489	0.06 ± 0.243
Item 6	1.00 ± 0.000	0.68 ± 0.476	0.45 ± 0.510	0.77 ± 0.514	0.68 ± 0.477	0.67 ± 0.492	0.81 ± 0.602	0.76 ± 0.436	0.72 ± 0.575	0.83 ± 0.388	0.40 ± 0.503	0.24 ± 0.437
Item 7	0.70 ± 0.675	0.82 ± 0.390	0.68 ± 0.477	0.85 ± 0.368	0.68 ± 0.477	0.42 ± 0.515	1.05 ± 0.218	0.90 ± 0.301	0.89 ± 0.323	0.57 ± 0.507	0.40 ± 0.503	0.18 ± 0.393
Item 8	0.20 ± 0.422	0.07 ± 0.262	0.00 ± 0.000	0.77 ± 0.430	0.59 ± 0.503	0.33 ± 0.492	1.05 ± 0.218	0.95 ± 0.218	0.83 ± 0.383	0.57 ± 0.507	0.10 ± 0.308	0.12 ± 0.332
Item 9	1.10 ± 0.568	0.86 ± 0.356	0.14 ± 0.351	0.77 ± 0.430	0.77 ± 0.429	0.58 ± 0.515	1.05 ± 0.218	1.00 ± 0.000	1.00 ± 0.000	0.87 ± 0.344	0.50 ± 0.513	0.59 ± 0.507
Item 10	1.00 ± 0.000	1.04 ± 0.189	1.00 ± 0.000	1.00 ± 0.000	1.00 ± 0.000	1.00 ± 0.000	1.10 ± 0.301	1.10 ± 0.301	1.00 ± 0.000	0.91 ± 0.288	0.60 ± 0.503	0.35 ± 0.493
Item 11	1.00 ± 0.000	0.93 ± 0.378	1.00 ± 0.000	0.96 ± 0.196	0.95 ± 0.213	0.92 ± 0.289	1.10 ± 0.301	1.14 ± 0.359	1.06 ± 0.236	0.96 ± 0.209	0.65 ± 0.489	0.29 ± 0.470
Item 12	0.70 ± 0.483	0.89 ± 0.315	0.77 ± 0.429	0.85 ± 0.543	0.86 ± 0.351	0.75 ± 0.452	0.76 ± 0.436	0.86 ± 0.478	0.61 ± 0.502	0.65 ± 0.487	0.45 ± 0.510	0.12 ± 0.332
Item 13	1.00 ± 0.000	0.89 ± 0.315	0.64 ± 0.492	0.88 ± 0.326	0.91 ± 0.294	0.83 ± 0.389	0.95 ± 0.218	0.95 ± 0.218	0.94 ± 0.236	0.78 ± 0.422	0.40 ± 0.503	0.24 ± 0.437
Item 14	0.20 ± 0.422	0.54 ± 0.508	0.36 ± 0.492	0.77 ± 0.430	0.86 ± 0.351	0.67 ± 0.492	1.00 ± 0.000	1.00 ± 0.316	0.83 ± 0.383	0.65 ± 0.487	0.35 ± 0.489	0.18 ± 0.393
Item 15	0.70 ± 0.483	0.46 ± 0.508	0.14 ± 0.351	0.50 ± 0.510	0.32 ± 0.477	0.17 ± 0.389	1.43 ± 0.676	1.19 ± 0.512	0.67 ± 0.485	0.30 ± 0.470	0.10 ± 0.308	0.41 ± 0.507
Item 16	0.40 ± 0.516	0.29 ± 0.460	0.27 ± 0.456	0.69 ± 0.471	0.45 ± 0.510	0.25 ± 0.452	0.95 ± 0.218	0.86 ± 0.359	0.61 ± 0.502	0.48 ± 0.511	0.00 ± 0.000	0.06 ± 0.243
Item 17	0.10 ± 0.316	0.14 ± 0.356	0.14 ± 0.351	0.08 ± 0.272	0.09 ± 0.294	0.00 ± 0.000	0.52 ± 0.512	0.33 ± 0.483	0.33 ± 0.485	0.30 ± 0.470	0.00 ± 0.000	0.12 ± 0.332

Abbreviations: F1: primary education/no education; F2: compulsory and noncompulsory secondary education and vocational training; F3: tertiary education/university study.

**Table A2:** Means and SDs for education level were obtained for the Hamilton scales for anxiety (Ham-Anx) and depression (Ham-Dep) after watching the video.

Scales/Items	Anxiety Disorder			Adjustment Disorder			Mixed Anxiety-Depressive Disorder			No Disorder		
HAM-ANX	F1	F2	F3	F1	F2	F3	F1	F2	F3	F1	F2	F3
Item 1	1.80 ± 0.632	1.54 ± 0.508	1.23 ± 0.429	1.08 ± 0.272	1.18 ± 0.395	1.08 ± 0.289	1.19 ± 0.402	1.33 ± 0.483	1.06 ± 0.236	1.00 ± 0.000	0.85 ± 0.489	0.41 ± 0.507
Item 2	1.50 ± 0.707	1.46 ± 0.637	1.05 ± 0.375	1.19 ± 0.402	1.23 ± 0.429	1.00 ± 0.000	1.19 ± 0.402	1.29 ± 0.463	1.17 ± 0.514	1.26 ± 0.541	0.55 ± 0.510	0.59 ± 0.618
Item 3	0.90 ± 0.994	0.54 ± 0.576	0.41 ± 0.503	0.46 ± 0.508	0.59 ± 0.666	0.17 ± 0.389	1.90 ± 0.436	1.76 ± 0.436	1.22 ± 0.647	0.61 ± 0.499	0.25 ± 0.444	0.00 ± 0.000
Item 4	2.10 ± 0.738	1.96 ± 0.189	1.27 ± 0.456	1.88 ± 0.326	1.91 ± 0.294	1.67 ± 0.492	2.00 ± 0.000	2.05 ± 0.218	1.67 ± 0.485	1.48 ± 0.511	1.15 ± 0.489	0.82 ± 0.393
Item 5	1.70 ± 0.823	1.43 ± 0.504	0.68 ± 0.477	1.35 ± 0.485	1.09 ± 0.684	1.00 ± 0.603	2.00 ± 0.000	1.90 ± 0.301	1.44 ± 0.511	1.09 ± 0.417	0.55 ± 0.510	0.24 ± 0.437
Item 6	1.20 ± 0.789	1.00 ± 0.720	0.23 ± 0.528	1.15 ± 0.543	1.09 ± 0.426	0.67 ± 0.492	1.95 ± 0.218	1.90 ± 0.301	1.22 ± 0.647	0.65 ± 0.573	0.05 ± 0.224	0.41 ± 0.507
Item 7	1.40 ± 0.516	1.43 ± 0.634	1.09 ± 0.294	1.38 ± 0.496	1.32 ± 0.568	0.92 ± 0.289	1.95 ± 0.218	1.95 ± 0.384	1.39 ± 0.502	1.13 ± 0.344	0.95 ± 0.224	0.71 ± 0.5888
Item 8	0.80 ± 0.919	0.54 ± 0.693	0.36 ± 0.492	0.35 ± 0.485	0.36 ± 0.492	0.08 ± 0.289	1.24 ± 0.539	0.57 ± 0.507	0.83 ± 0.514	0.91 ± 0.417	0.30 ± 0.470	0.18 ± 0.393
Item 9	2.10 ± 0.316	1.86 ± 0.356	1.18 ± 0.588	1.58 ± 0.504	1.77 ± 0.429	1.67 ± 0.492	1.86 ± 0.573	2.00 ± 0.447	1.56 ± 0.705	1.61 ± 0.583	1.20 ± 0.410	1.00 ± 0.000
Item 10	2.20 ± 0.422	2.07 ± 0.262	1.68 ± 0.568	1.73 ± 0.452	1.55 ± 0.596	1.33 ± 0.492	1.52 ± 0.512	1.71 ± 0.463	1.61 ± 0.502	1.61 ± 0.499	1.20 ± 0.410	0.71 ± 0.470
Item 11	2.20 ± 0.422	1.93 ± 0.378	1.64 ± 0.581	1.73 ± 0.452	1.41 ± 0.590	1.25 ± 0.622	1.81 ± 0.402	1.76 ± 0.436	1.50 ± 0.514	1.13 ± 0.344	1.00 ± 0.324	0.65 ± 0.493
Item 12	1.40 ± 0.966	0.79 ± 0.686	0.95 ± 0.486	1.12 ± 0.711	0.59 ± 0.734	0.75 ± 0.622	1.10 ± 0.436	1.05 ± 0.218	1.00 ± 0.594	0.96 ± 0.475	0.80 ± 0.410	0.53 ± 0.514
Item 13	2.10 ± 0.316	1.82 ± 0.390	1.59 ± 0.590	1.46 ± 0.647	1.09 ± 0.684	1.25 ± 0.452	1.19 ± 0.512	1.29 ± 0.561	1.00 ± 0.594	0.96 ± 0.209	0.85 ± 0.366	0.65 ± 0.493
Item 14	2.00 ± 0.667	1.71 ± 0.535	1.18 ± 0.395	1.54 ± 0.508	1.82 ± 0.395	1.50 ± 0.522	2.67 ± 0.483	2.67 ± 0.483	2.00 ± 0.767	1.65 ± 0.487	1.20 ± 0.696	0.88 ± 0.485
HRS-D												
Item 1	1.00 ± 0.667	0.86 ± 0.591	0.68 ± 0.568	0.96 ± 0.344	0.86 ± 0.468	0.83 ± 0.389	1.19 ± 0.402	1.29 ± 0.463	1.17 ± 0.383	0.74 ± 0.449	0.20 ± 0.410	0.00 ± 0.000
Item 2	1.40 ± 0.699	1.07 ± 0.716	0.86 ± 0.351	1.00 ± 0.283	0.86 ± 0.468	0.67 ± 0.492	1.10 ± 0.301	1.14 ± 0.359	1.11 ± 0.323	0.26 ± 0.449	0.20 ± 0.410	0.12 ± 0.332
Item 3	0.30 ± 0.483	0.11 ± 0.315	0.00 ± 0.000	0.27 ± 0.452	0.14 ± 0.351	0.00 ± 0.000	0.95 ± 0.218	0.86 ± 0.359	0.50 ± 0.514	0.17 ± 0.388	0.05 ± 0.224	0.00 ± 0.000
Item 4	1.80 ± 0.422	1.46 ± 0.508	1.09 ± 0.294	1.77 ± 0.430	1.64 ± 0.492	1.50 ± 0.522	1.86 ± 0.359	2.00 ± 0.316	1.83 ± 0.383	1.30 ± 0.559	1.15 ± 0.366	0.76 ± 0.437
Item 5	1.70 ± 0.483	1.54 ± 0.508	0.77 ± 0.685	1.35 ± 0.629	1.59 ± 0.503	1.33 ± 0.492	1.95 ± 0.218	2.00 ± 0.000	1.72 ± 0.461	1.35 ± 0.487	0.90 ± 0.553	0.65 ± 0.493
Item 6	1.50 ± 0.707	1.18 ± 0.612	0.77 ± 0.612	1.15 ± 0.732	1.41 ± 0.734	1.58 ± 0.515	1.95 ± 0.218	2.00 ± 0.000	1.67 ± 0.594	1.22 ± 0.600	0.90 ± 0.447	0.71 ± 0.470
Item 7	0.90 ± 0.738	0.86 ± 0.356	0.91 ± 0.294	1.08 ± 0.392	1.00 ± 0.535	1.00 ± 0.426	1.90 ± 0.301	1.81 ± 0.402	1.22 ± 0.548	0.61 ± 0.499	0.35 ± 0.489	0.18 ± 0.393
Item 8	0.40 ± 0.843	0.25 ± 0.441	0.09 ± 0.294	1.08 ± 0.484	0.73 ± 0.631	0.67 ± 0.651	1.38 ± 0.498	1.24 ± 0.539	1.00 ± 0.343	0.61 ± 0.499	0.10 ± 0.308	0.12 ± 0.332
Item 9	1.30 ± 0.823	1.36 ± 0.621	0.91 ± 0.610	1.04 ± 0.528	0.91 ± 0.426	0.83 ± 0.389	1.38 ± 0.669	1.38 ± 0.590	1.17 ± 0.383	0.83 ± 0.388	0.70 ± 0.470	0.65 ± 0.493
Item 10	2.00 ± 0.000	1.96 ± 0.331	1.59 ± 0.503	1.35 ± 0.562	1.45 ± 0.510	1.17 ± 0.389	1.48 ± 0.680	1.52 ± 0.602	1.11 ± 0.471	0.91 ± 0.288	0.75 ± 0.444	0.47 ± 0.514
Item 11	1.90 ± 0.316	1.89 ± 0.315	1.27 ± 0.456	1.46 ± 0.582	1.59 ± 0.503	1.58 ± 0.515	1.86 ± 0.359	1.86 ± 0.359	1.50 ± 0.514	1.00 ± 0.302	1.00 ± 0.459	0.41 ± 0.507
Item 12	1.40 ± 0.516	1.18 ± 0.390	1.00 ± 0.436	1.19 ± 0.567	1.05 ± 0.375	0.92 ± 0.289	1.14 ± 0.359	1.14 ± 0.359	1.11 ± 0.323	0.78 ± 0.422	0.70 ± 0.470	0.47 ± 0.514
Item 13	1.20 ± 0.422	1.25 ± 0.441	1.05 ± 0.375	0.96 ± 0.445	1.18 ± 0.501	1.08 ± 0.289	1.19 ± 0.402	1.24 ± 0.436	1.11 ± 0.323	0.87 ± 0.344	0.70 ± 0.470	0.65 ± 0.493
Item 14	0.90 ± 0.738	0.64 ± 0.559	0.59 ± 0.503	0.96 ± 0.445	1.00 ± 0.436	1.00 ± 0.000	1.05 ± 0.218	1.00 ± 0.316	1.06 ± 0.236	0.87 ± 0.344	0.60 ± 0.681	0.35 ± 0.493
Item 15	0.60 ± 0.516	0.36 ± 0.488	0.09 ± 0.294	0.81 ± 0.491	0.41 ± 0.503	0.17 ± 0.389	1.57 ± 0.507	1.57 ± 0.507	1.06 ± 0.639	0.43 ± 0.507	0.25 ± 0.444	0.35 ± 0.493
Item 16	0.50 ± 0.527	0.32 ± 0.548	0.55 ± 0.510	1.00 ± 0.400	0.73 ± 0.456	0.58 ± 0.515	1.48 ± 0.602	1.33 ± 0.577	1.06 ± 0.725	0.48 ± 0.511	0.10 ± 0.308	0.00 ± 0.000
Item 17	0.30 ± 0.483	0.25 ± 0.441	0.23 ± 0.429	0.15 ± 0.368	0.18 ± 0.395	0.17 ± 0.389	1.10 ± 0.436	1.10 ± 0.301	0.83 ± 0.514	0.26 ± 0.449	0.05 ± 0.224	0.12 ± 0.332

Abbreviations: F1: primary education/no education; F2: compulsory and noncompulsory secondary education and vocational training; F3: tertiary education/university study.

**Table A3:** Means and SDs for education level were obtained for the Hamilton scales for anxiety (Ham-Anx) and depression (Ham-Dep) seven days after watching the video.

Scales/Items	Anxiety Disorder			Adjustment Disorder			Mixed Anxiety-Depressive Disorder			No Disorder		
HAM-A	F1	F2	F3	F1	F2	F3	F1	F2	F3	F1	F2	F3
Item 1	1.60 ± 0.699	1.04 ± 0.331	0.95 ± 0.375	1.00 ± 0.000	1.00 ± 0.000	1.00 ± 0.000	1.19 ± 0.402	1.19 ± 0.402	1.06 ± 0.416	0.96 ± 0.209	0.90 ± 0.447	0.06 ± 0.243
Item 2	1.20 ± 0.422	1.00 ± 0.471	0.86 ± 0.351	1.04 ± 0.196	1.00 ± 0.000	0.92 ± 0.289	1.33 ± 0.483	1.29 ± 0.463	1.06 ± 0.236	0.83 ± 0.388	0.55 ± 0.510	0.53 ± 0.514
Item 3	0.70 ± 0.949	0.39 ± 0.567	0.14 ± 0.351	0.42 ± 0.578	0.27 ± 0.456	0.17 ± 0.389	1.57 ± 0.676	1.29 ± 0.644	0.78 ± 0.732	0.48 ± 0.511	0.15 ± 0.366	0.00 ± 0.000
Item 4	1.70 ± 0.675	1.57 ± 0.573	1.05 ± 0.375	1.42 ± 0.504	1.36 ± 0.492	1.17 ± 0.389	2.00 ± 0.000	1.86 ± 0.359	1.50 ± 0.514	1.35 ± 0.487	0.95 ± 0.224	0.71 ± 0.470
Item 5	1.30 ± 0.675	0.93 ± 0.604	0.59 ± 0.503	1.00 ± 0.632	0.95 ± 0.375	0.75 ± 0.452	1.90 ± 0.301	1.67 ± 0.483	1.22 ± 0.428	0.96 ± 0.209	0.45 ± 0.510	0.12 ± 0.332
Item 6	0.90 ± 0.316	0.68 ± 0.548	0.27 ± 0.456	0.96 ± 0.599	0.86 ± 0.560	0.92 ± 0.289	1.76 ± 0.436	1.57 ± 0.507	0.89 ± 0.676	0.39 ± 0.499	0.00 ± 0.000	0.18 ± 0.393
Item 7	1.00 ± 0.471	1.07 ± 0.539	0.82 ± 0.501	0.88 ± 0.326	1.09 ± 0.426	0.83 ± 0.389	1.76 ± 0.436	1.62 ± 0.590	1.00 ± 0.594	1.00 ± 0.426	0.65 ± 0.489	0.59 ± 0.507
Item 8	0.30 ± 0.483	0.14 ± 0.356	0.32 ± 0.477	0.27 ± 0.452	0.18 ± 0.501	0.08 ± 0.289	0.86 ± 0.727	0.48 ± 0.512	0.72 ± 0.461	0.91 ± 0.417	0.20 ± 0.410	0.24 ± 0.437
Item 9	1.50 ± 0.527	1.43 ± 0.573	0.91 ± 0.294	1.19 ± 0.491	1.27 ± 0.550	1.17 ± 0.389	1.86 ± 0.573	1.90 ± 0.539	1.44 ± 0.616	1.09 ± 0.417	1.20 ± 0.410	0.94 ± 0.243
Item 10	1.90 ± 0.568	1.75 ± 0.441	1.05 ± 0.375	1.19 ± 0.402	1.32 ± 0.477	1.08 ± 0.289	1.38 ± 0.498	1.67 ± 0.483	1.33 ± 0.485	1.00 ± 0.000	1.00 ± 0.459	0.76 ± 0.437
Item 11	1.90 ± 0.738	1.68 ± 0.476	0.95 ± 0.375	1.23 ± 0.587	1.14 ± 0.640	1.17 ± 0.389	1.67 ± 0.483	1.71 ± 0.463	1.28 ± 0.461	1.00 ± 0.426	0.50 ± 0.607	0.47 ± 0.514
Item 12	0.70 ± 0.823	0.57 ± 0.634	0.73 ± 0.456	0.73 ± 0.667	0.45 ± 0.596	0.75 ± 0.622	1.00 ± 0.447	0.67 ± 0.483	0.72 ± 0.461	0.87 ± 0.344	0.40 ± 0.503	0.29 ± 0.470
Item 13	1.70 ± 0.483	1.32 ± 0.670	0.91 ± 0.426	1.00 ± 0.632	0.91 ± 0.684	0.67 ± 0.492	1.29 ± 0.463	1.19 ± 0.512	0.78 ± 0.647	0.61 ± 0.499	0.50 ± 0.513	0.35 ± 0.493
Item 14	1.30 ± 0.823	1.25 ± 0.585	0.68 ± 0.477	1.15 ± 0.464	1.36 ± 0.492	1.25 ± 0.452	2.19 ± 0.512	2.19 ± 0.512	1.56 ± 0.705	1.04 ± 0.367	1.00 ± 0.562	0.59 ± 0.507
**HAM-D**												
Item 1	0.90 ± 0.316	0.57 ± 0.504	0.32 ± 0.477	0.96 ± 0.196	0.82 ± 0.395	0.67 ± 0.492	1.10 ± 0.301	1.10 ± 0.301	1.06 ± 0.236	0.74 ± 0.449	0.20 ± 0.410	0.00 ± 0.000
Item 2	1.20 ± 0.632	0.61 ± 0.629	0.73 ± 0.456	0.77 ± 0.430	0.82 ± 0.395	0.67 ± 0.492	1.05 ± 0.218	1.00 ± 0.000	1.00 ± 0.000	0.22 ± 0.422	0.20 ± 0.410	0.06 ± 0.243
Item 3	0.30 ± 0.483	0.11 ± 0.315	0.00 ± 0.000	0.23 ± 0.430	0.09 ± 0.294	0.08 ± 0.289	0.86 ± 0.478	0.76 ± 0.436	0.56 ± 0.511	0.17 ± 0.388	0.00 ± 0.000	0.00 ± 0.000
Item 4	1.30 ± 0.483	1.18 ± 0.612	0.86 ± 0.351	1.31 ± 0.549	1.32 ± 0.477	1.08 ± 0.289	1.81 ± 0.402	1.76 ± 0.436	1.50 ± 0.514	1.04 ± 0.209	0.95 ± 0.224	0.71 ± 0.470
Item 5	1.00 ± 0.471	1.18 ± 0.548	0.95 ± 0.213	0.96 ± 0.599	1.14 ± 0.351	1.00 ± 0.426	1.81 ± 0.402	1.86 ± 0.478	1.28 ± 0.669	1.04 ± 0.209	0.65 ± 0.489	0.35 ± 0.493
Item 6	1.10 ± 0.568	0.86 ± 0.448	0.68 ± 0.477	0.81 ± 0.567	0.86 ± 0.468	0.83 ± 0.389	1.71 ± 0.561	1.81 ± 0.512	1.00 ± 0.840	0.78 ± 0.518	0.55 ± 0.686	0.29 ± 0.470
Item 7	0.90 ± 0.568	0.54 ± 0.508	0.73 ± 0.456	0.85 ± 0.464	0.59 ± 0.503	0.25 ± 0.452	1.43 ± 0.598	1.52 ± 0.512	0.94 ± 0.725	0.48 ± 0.511	0.25 ± 0.444	0.29 ± 0.470
Item 8	0.00 ± 0.000	0.14 ± 0.356	0.00 ± 0.000	0.88 ± 0.588	0.50 ± 0.512	0.42 ± 0.515	1.05 ± 0.384	1.19 ± 0.512	0.89 ± 0.583	0.52 ± 0.511	0.10 ± 0.308	0.12 ± 0.332
Item 9	0.70 ± 0.483	0.79 ± 0.568	0.41 ± 0.503	0.77 ± 0.430	0.73 ± 0.456	0.75 ± 0.452	1.05 ± 0.218	1.05 ± 0.384	1.06 ± 0.416	0.74 ± 0.449	0.50 ± 0.513	0.59 ± 0.507
Item 10	1.80 ± 0.422	1.50 ± 0.509	1.00 ± 0.000	1.15 ± 0.368	1.05 ± 0.213	1.08 ± 0.289	1.24 ± 0.436	1.29 ± 0.463	1.11 ± 0.323	0.91 ± 0.288	0.60 ± 0.503	0.41 ± 0.507
Item 11	1.80 ± 0.422	1.50 ± 0.509	1.00 ± 0.000	1.12 ± 0.431	1.14 ± 0.351	1.00 ± 0.000	1.76 ± 0.436	1.76 ± 0.436	1.33 ± 0.594	0.96 ± 0.209	0.65 ± 0.489	0.53 ± 0.514
Item 12	1.20 ± 0.632	1.04 ± 0.508	0.86 ± 0.351	0.85 ± 0.464	0.82 ± 0.395	0.92 ± 0.289	1.14 ± 0.478	1.33 ± 0.483	1.00 ± 0.485	0.78 ± 0.422	0.45 ± 0.510	0.06 ± 0.243
Item 13	0.90 ± 0.568	0.93 ± 0.466	0.82 ± 0.395	0.81 ± 0.402	0.82 ± 0.395	0.83 ± 0.389	1.14 ± 0.359	1.19 ± 0.512	1.00 ± 0.000	0.87 ± 0.344	0.60 ± 0.503	0.24 ± 0.437
Item 14	0.60 ± 0.516	0.57 ± 0.504	0.45 ± 0.510	0.62 ± 0.496	0.59 ± 0.503	0.58 ± 0.515	1.05 ± 0.498	1.05 ± 0.498	0.83 ± 0.383	0.57 ± 0.507	0.35 ± 0.489	0.18 ± 0.393
Item 15	0.60 ± 0.516	0.32 ± 0.476	0.05 ± 0.213	0.31 ± 0.471	0.23 ± 0.429	0.25 ± 0.452	1.05 ± 0.740	1.05 ± 0.590	0.61 ± 0.608	0.26 ± 0.449	0.15 ± 0.366	0.41 ± 0.507
Item 16	0.50 ± 0.527	0.18 ± 0.390	0.18 ± 0.395	0.62 ± 0.496	0.32 ± 0.477	0.08 ± 0.289	0.67 ± 0.577	0.38 ± 0.498	0.28 ± 0.461	0.35 ± 0.487	0.00 ± 0.000	0.06 ± 0.243
Item 17	0.80 ± 0.422	0.21 ± 0.418	0.18 ± 0.395	0.12 ± 0.326	0.09 ± 0.294	0.17 ± 0.389	1.00 ± 0.548	0.76 ± 0.539	0.50 ± 0.514	0.26 ± 0.449	0.05 ± 0.224	0.12 ± 0.332

Abbreviations: F1: primary education/no education; F2: compulsory and noncompulsory secondary education and vocational training; F3: tertiary education/university study.

**Table A4:** Means and SDs of education level obtained on the MDAS and DFS seven days after watching the video.

Scales/Items	Anxiety Disorder			Adjustment Disorder			Mixed Anxiety-Depressive Disorder			No Disorder		
MDAS	F1	F2	F3	F1	F2	F3	F1	F2	F3	F1	F2	F3
Item 1	1.80 ± 0.422	1.21 ± 0.499	1.00 ± 0.00	1.85 ± 0.464	1.91 ± 0.294	1.67 ± 0.492	2.67 ± 0.577	2.43 ± 0.507	2.11 ± 0.323	1.91 ± 0.288	1.45 ± 0.510	1.12 ± 0.332
Item 2	1.80 ± 0.422	1.32 ± 0.548	1.14 ± 0.351	1.92 ± 0.392	1.86 ± 0.351	1.50 ± 0.522	2.67 ± 0.577	2.38 ± 0.498	2.11 ± 0.323	2.00 ± 0.000	1.50 ± 0.513	1.12 ± 0.332
Item 3	3.00 ± 0.667	1.96 ± 0.576	1.55 ± 0.510	2.12 ± 0.326	2.00 ± 0.309	2.00 ± 0.000	2.76 ± 0.700	2.48 ± 0.512	2.22 ± 0.732	2.09 ± 0.288	1.65 ± 0.489	1.29 ± 0.470
Item 4	2.30 ± 0.483	1.64 ± 0.559	1.14 ± 0.351	1.77 ± 0.514	1.82 ± 0.588	1.42 ± 0.515	2.76 ± 0.700	2.48 ± 0.602	2.17 ± 0.618	1.91 ± 0.417	1.50 ± 0.513	1.24 ± 0.437
Item 5	2.80 ± 0.422	2.00 ± 0.471	1.41 ± 0.503	2.12 ± 0.431	2.05 ± 0.375	1.83 ± 0.577	2.86 ± 0.655	2.52 ± 0.512	2.28 ± 0.575	2.04 ± 0.209	1.65 ± 0.587	1.29 ± 0.470
**DFS**												
Item 1	1.30 ± 0.483	1.29 ± 0.460	1.00 ± 0.000	1.96 ± 0.774	2.00 ± 0.816	1.50 ± 0.674	2.95 ± 0.218	2.62 ± 0.669	2.39 ± 0.778	1.91 ± 0.515	1.25 ± 0.444	1.12 ± 0.332
Item 2	1.50 ± 0.527	1.29 ± 0.460	1.00 ± 0.000	1.92 ± 0.744	1.91 ± 0.750	1.50 ± 0.674	2.86 ± 0.359	2.62 ± 0.669	2.28 ± 0.752	1.96 ± 0.367	1.25 ± 0.444	1.00 ± 0.000
Item 3	2.30 ± 0.675	1.64 ± 0.678	1.32 ± 0.477	2.08 ± 0.560	2.18 ± 0.501	1.75 ± 0.754	2.90 ± 0.436	2.81 ± 0.402	2.50 ± 0.514	2.04 ± 0.209	1.65 ± 0.671	1.18 ± 0.393
Item 4	2.30 ± 0.675	1.46 ± 0.637	1.14 ± 0.351	2.08 ± 0.560	1.91 ± 0.610	1.58 ± 0.669	2.81 ± 0.512	2.76 ± 0.436	2.44 ± 0.616	2.09 ± 0.2888	1.65 ± 0.745	1.18 ± 0.393
Item 5	2.20 ± 0.632	1.39 ± 0.567	1.14 ± 0.351	2.04 ± 0.662	1.95 ± 0.575	1.67 ± 0.492	2.81 ± 0.750	2.71 ± 0.561	2.28 ± 0.669	2.04 ± 0.367	1.30 ± 0.470	1.12 ± 0.332
Item 6	2.10 ± 0.738	1.32 ± 0.476	1.41 ± 1.098	1.81 ± 0.749	1.77 ± 0.612	1.58 ± 0.515	2.90 ± 0.944	2.67 ± 0.730	2.28 ± 0.669	2.04 ± 0.367	1.25 ± 0.444	1.00 ± 0.000
Item 7	2.70 ± 0.675	1.68 ± 0.548	1.36 ± 0.492	2.12 ± 0.516	2.09 ± 0.426	2.00 ± 0.426	3.05 ± 0.805	2.76 ± 0.625	2.44 ± 0.616	2.17 ± 0.388	1.65 ± 0.671	1.29 ± 0.470
Item 8	1.90 ± 0.568	1.25 ± 0.518	1.00 ± 0.000	1.65 ± 0.745	2.05 ± 0.785	1.67 ± 0.778	3.05 ± 0.590	2.76 ± 0.768	2.72 ± 0.752	1.96 ± 0.562	1.45 ± 0.605	1.12 ± 0.332
Item 9	2.00 ± 0.667	1.21 ± 0.499	1.00 ± 0.000	1.69 ± 0.838	1.95 ± 0.722	1.58 ± 0.793	2.90 ± 0.625	2.71 ± 0.845	2.78 ± 0.647	1.96 ± 0.475	1.55 ± 0.605	1.06 ± 0.243
Item 10	2.40 ± 0.699	1.46 ± 0.576	1.05 ± 0.213	1.69 ± 0.736	1.95 ± 0.722	1.67 ± 0.778	2.90 ± 0.625	2.76 ± 0.768	2.72 ± 0.752	1.87 ± 0.458	1.50 ± 0.688	1.06 ± 0.243
Item 11	3.20 ± 0.789	2.11 ± 0.737	1.64 ± 0.492	2.23 ± 0.863	2.59 ± 0.734	2.42 ± 0.669	3.76 ± 0.700	3.33 ± 0.796	3.06 ± 0.802	2.61 ± 0.583	1.70 ± 0.801	1.29 ± 0.470
Item 12	2.50 ± 1.080	1.61 ± 0.737	1.23 ± 0.429	1.92 ± 0.935	2.14 ± 0.640	1.75 ± 0.754	3.33 ± 0.577	3.10 ± 0.831	2.83 ± 0.618	2.13 ± 0.548	1.65 ± 0.813	1.06 ± 0.243
Item 13	2.90 ± 0.876	1.93 ± 0.766	1.32 ± 0.477	1.92 ± 0.977	2.14 ± 0.710	1.75 ± 0.754	3.71 ± 0.644	3.24 ± 1.044	3.06 ± 0.725	2.13 ± 0.626	1.75 ± 0.910	1.59 ± 2.425
Item 14	2.40 ± 0.966	1.43 ± 0.573	1.14 ± 0.351	1.88 ± 0.816	2.05 ± 0.785	1.83 ± 0.718	3.48 ± 0.602	3.00 ± 1.049	3.22 ± 0.548	2.43 ± 0.590	1.85 ± 0.813	1.41 ± 0.507
Item 15	3.60 ± 0.699	2.32 ± 0.905	1.82 ± 0.395	2.54 ± 0.905	2.77 ± 0.685	2.42 ± 0.669	3.95 ± 0.498	3.48 ± 0.873	3.39 ± 0.608	2.91 ± 0.417	2.10 ± 0.718	1.88 ± 0.332
Item 16	2.40 ± 1.174	1.14 ± 0.448	1.05 ± 0.213	1.65 ± 0.797	1.91 ± 0.750	1.42 ± 0.793	3.29 ± 0.717	2.86 ± 0.964	3.06 ± 0.639	2.35 ± 0.714	1.55 ± 0.686	1.41 ± 0.507
Item 17	2.60 ± 1.174	1.39 ± 0.685	1.09 ± 0.294	1.85 ± 0.784	2.00 ± 0.756	1.50 ± 0.798	3.52 ± 0.814	2.95 ± 0.921	3.11 ± 0.676	2.57 ± 0.662	1.65 ± 0.745	1.53 ± 0.514
Item 18	3.60 ± 0.843	2.43 ± 0.742	1.95 ± 0.213	2.58 ± 0.758	2.77 ± 0.685	2.42 ± 0.669	3.90 ± 0.625	3.67 ± 0.730	3.50 ± 0.786	2.96 ± 0.475	2.30 ± 0.733	1.88 ± 0.332
Item 19	2.00 ± 0.943	1.43 ± 0.573	1.14 ± 0.351	1.62 ± 0.697	1.91 ± 0.811	1.58 ± 0.515	3.33 ± 0.796	2.90 ± 0.889	2.56 ± 1.042	1.43 ± 0.507	1.45 ± 0.759	1.00 ± 0.000
Item 20	3.30 ± 0.823	2.14 ± 0.848	1.41 ± 0.503	2.42 ± 0.809	2.45 ± 0.596	1.92 ± 0.669	3.95 ± 0.498	3.52 ± 0.814	3.33 ± 0.686	2.74 ± 0.449	2.10 ± 0.788	1.47 ± 0.514
Item 21	3.30 ± 0.823	2.14 ± 0.848	1.36 ± 0.492	2.42 ± 0.809	2.50 ± 0.598	1.92 ± 0.669	3.95 ± 0.498	3.57 ± 0.811	3.33 ± 0.686	2.78 ± 0.422	2.10 ± 0.788	1.65 ± 0.493
Item 22	3.30 ± 0.949	1.96 ± 0.693	1.18 ± 0.395	2.50 ± 0.860	2.73 ± 0.767	2.25 ± 0.866	3.67 ± 0.483	3.67 ± 0.483	3.39 ± 0.608	3.26 ± 0.449	1.90 ± 1.119	1.24 ± 0.562
Item 23	3.30 ± 0.949	1.96 ± 0.693	1.18 ± 0.395	2.50 ± 0.860	2.64 ± 0.727	2.33 ± 0.985	3.67 ± 0.483	3.67 ± 0.483	3.39 ± 0.608	3.22 ± 0.518	1.85 ± 1.089	1.24 ± 0.562
Item 24	3.30 ± 0.823	1.96 ± 0.693	1.23 ± 0.429	2.50 ± 0.860	2.59 ± 0.854	2.33 ± 0.778	3.62 ± 0.590	3.57 ± 0.598	3.39 ± 0.608	3.22 ± 0.518	1.85 ± 1.089	1.24 ± 0.562

Abbreviations: F1: primary education/no education; F2: compulsory and noncompulsory secondary education and vocational training; F3: tertiary education/university study.

**Table A5:** Means and SDs of education level obtained on the MDAS and DFS scales after watching the video.

Scales/Items	Anxiety Disorder			Adjustment Disorder			Mixed Anxiety-Depressive Disorder			No Disorder		
MDAS	F1	F2	F3	F1	F2	F3	F1	F2	F3	F1	F2	F3
Item 1	3.00 ± 1.247	2.43 ± 0.742	1.68 ± 0.568	3.00 ± 0.566	3.09 ± 0.294	2.83 ± 0.577	3.90 ± 0.301	3.95 ± 0.498	3.22 ± 0.732	2.74 ± 0.541	1.95 ± 0.945	1.53 ± 0.717
Item 2	3.10 ± 0.994	2.54 ± 0.637	1.73 ± 0.550	3.00 ± 0.566	3.05 ± 0.375	2.83 ± 0.577	4.05 ± 0.384	4.00 ± 0.548	3.28 ± 0.752	2.78 ± 0.422	1.90 ± 0.912	1.65 ± 0.702
Item 3	3.60 ± 0.843	3.25 ± 0.585	2.27 ± 0.456	3.15 ± 0.675	3.32 ± 0.646	3.08 ± 0.515	4.52 ± 0.680	4.62 ± 0.590	3.44 ± 0.984	2.83 ± 0.388	1.90 ± 0.852	1.47 ± 0.624
Item 4	3.30 ± 0.823	2.46 ± 0.576	1.64 ± 0.492	2.85 ± 0.881	2.91 ± 0.610	2.75 ± 0.622	4.38 ± 0.590	3.44 ± 0.984	3.39 ± 1.037	2.74 ± 0.449	1.95 ± 0.887	1.59 ± 0.618
Item 5	360 ± 0.699	3.21 ± 0.630	2.64 ± 1.706	3.27 ± 0.874	3.36 ± 0.727	2.92 ± 0.515	4.81 ± 0.402	4.67 ± 0.577	3.50 ± 0.985	2.83 ± 0.388	2.05 ± 0.999	1.76 ± 0.752
**DFS**												
Item 1	1.80 ± 0.422	1.89 ± 0.629	1.05 ± 0.213	2.62 ± 0.637	2.59 ± 0.796	2.25 ± 0.754	2.95 ± 0.218	3.14 ± 0.655	2.44 ± 0.784	1.91 ± 0.515	1.25 ± 0.444	1.12 ± 0.332
Item 2	3.90 ± 6.367	1.93 ± 0.716	1.05 ± 0.213	2.58 ± 0.703	2.59 ± 0.796	2.17 ± 0.718	2.90 ± 0.301	3.14 ± 0.727	2.39 ± 0.778	2.00 ± 0.426	1.25 ± 0.444	1.00 ± 0.354
Item 3	3.30 ± 1.059	2.86 ± 0.803	1.95 ± 0.653	3.04 ± 0.774	3.32 ± 0.716	3.00 ± 0.603	4.33 ± 0.730	4.19 ± 0.750	3.28 ± 0.958	2.57 ± 0.507	1.85 ± 0.813	1.53 ± 0.717
Item 4	3.60 ± 0.843	2.86 ± 0.591	1.86 ± 0.468	3.23 ± 0.863	3.41 ± 0.734	3.00± 0.603	4.48 ± 0.602	4.52 ± 0.680	3.56 ± 0.984	2.57 ± 0.590	2.05 ± 0.945	1.59 ± 0.712
Item 5	3.50 ± 0.850	2.64 ± 0.621	1.77 ± 0.612	3.23 ± 0.863	3.23 ± 0.685	2.92 ± 0.669	4.57 ± 0.507	6.81 ± 11.062	3.39 ± 0.979	2.65 ± 0.487	1.70 ± 0.801	1.41 ± 0.507
Item 6	3.50 ± 0.850	2.68 ± 0.863	1.68 ± 0.716	3.00 ± 0.894	3.14 ± 0.774	2.83 ± 0.718	4.57 ± 0.507	4.33 ± 0.730	3.50 ± 0.985	2.65 ± 0.487	1.75 ± 0.851	1.24 ± 0.437
Item 7	3.50 ± 0.972	2.96 ± 0.744	1.86 ± 0.468	3.50 ± 0.990	3.59 ± 0.666	3.08 ± 0.793	4.86 ± 0.359	4.67 ± 0.730	3.78 ± 0.943	2.83 ± 0.388	2.15 ± 0.988	1.65 ± 0.606
Item 8	2.40 ± 0.843	2.00 ± 0.943	1.14 ± 0.351	2.46 ± 0.859	3.05 ± 0.486	2.75 ± 0.622	3.90 ± 0.436	3.71 ± 0.463	3.44 ± 0.784	2.39 ± 0.783	1.90 ± 1.071	1.29 ± 0.470
Item 9	2.50 ± 0.707	2.21 ± 0.833	1.14 ± 0.351	2.50 ± 0.906	3.09 ± 0.526	2.75 ± 0.622	3.95 ± 0.498	3.71 ± 0.463	3.44 ± 0.705	2.39 ± 0.783	1.95 ± 1.050	1.35 ± 0.493
Item 10	3.10 ± 0.568	2.50 ± 0.793	1.55 ± 0.596	2.42 ± 0.857	2.95 ± 0.722	2.83 ± 0.718	3.95 ± 0.498	3.81 ± 0.602	3.44 ± 0.856	2.48 ± 0.730	2.00 ± 1.026	1.41 ± 0.618
Item 11	4.20 ± 0.632	3.50 ± 0.793	2.41 ± 0.503	3.31 ± 0.884	3.68 ± 0.646	3.42 ± 0.515	4.33 ± 0.483	4.29 ± 0.644	3.83 ± 0.857	2.96 ± 0.475	2.45 ± 1.099	1.65 ± 0.702
Item 12	3.40 ± 0.843	2.79 ± 0.738	1.77 ± 0.685	3.12 ± 0.766	3.32 ± 0.716	3.00 ± 0.853	4.19 ± 0.512	4.38 ± 0.669	3.67 ± 0.907	2.70 ± 0.703	0.20 ± 1.056	1.41 ± 0.507
Item 13	4.00 ± 0.816	3.32 ± 0.723	1.86 ± 0.834	3.04 ± 0.999	3.36 ± 0.790	3.00 ± 0.953	4.62 ± 0.498	4.52 ± 0.602	3.94 ± 0.873	3.00 ± 0.739	2.30 ± 1.218	1.53 ± 0.624
Item 14	2.80 ± 1.033	2.46 ± 0.838	1.36 ± 0.658	2.54 ± 0.905	3.00 ± 0.690	2.75 ± 0.622	4.67 ± 0.483	4.19 ± 0.814	3.78 ± 0.647	3.22 ± 0.600	2.60 ± 1.046	1.82 ± 0.529
Item 15	4.60 ± 0.516	3.82 ± 0.476	2.64 ± 0.658	3.81 ± 0.895	4.14 ± 0.710	3.42 ± 0.515	4.90 ± 0.301	4.90 ± 0.301	4.50 ± 0.857	3.48 ± 0.593	2.90 ± 0.788	2.53 ± 0.514
Item 16	2.70 ± 1.337	2.18 ± 0.723	1.14 ± 0.468	2.46 ± 0.811	2.68 ± 0.716	2.42 ± 0.515	4.19 ± 0.512	3.95 ± 0.590	3.67 ± 0.485	3.00 ± 0.798	2.30 ± 1.081	1.53 ± 0.514
Item 17	3.20 ± 0.789	2.46 ± 0.838	1.32 ± 0.646	2.65 ± 0.936	2.86 ± 0.774	2.33 ± 0.651	4.10 ± 0.539	4.10 ± 0.625	3.78 ± 0.548	3.17 ± 0.717	2.55 ± 0.826	1.76 ± 0.664
Item 18	4.40 ± 0.699	3.79 ± 0.499	2.73 ± 0.631	3.77 ± 0.951	3.95 ± 0.844	3.50 ± 0.522	4.81 ± 0.402	4.76 ± 0.539	4.33 ± 0.840	3.43 ± 0.507	2.75 ± 0.716	2.24 ± 0.437
Item 19	2.60 ± 1.174	2.21 ± 0.957	1.82 ± 0.588	2.27 ± 1.041	2.68 ± 0.945	2.42 ± 0.900	3.81 ± 0.750	4.05 ± 0.590	3.06 ± 1.305	2.09 ± 1.164	1.70 ± 1.081	1.18 ± 0.393
Item 20	4.00 ± 0.471	3.71 ± 0.460	2.64 ± 0.658	3.65 ± 0.892	3.73 ± 0.767	3.08 ± 0.900	5.00 ± 0.000	7.29 ± 10.937	4.44 ± 0.784	3.70 ± 0.470	2.90 ± 0.852	2.29 ± 0.588
Item 21	4.00 ± 0.471	3.71 ± 0.460	2.59 ± 0.666	3.69 ± 0.928	3.82 ± 0.795	3.08 ± 0.900	5.00 ± 0.00	4.86 ± 0.359	4.50 ± 0.786	3.70 ± 0.470	2.80 ± 0.768	2.41 ± 0.507
Item 22	3.50 ± 0.707	2.71 ± 0.600	1.41 ± 0.503	3.19 ± 0.849	3.55 ± 0.510	3.08 ± 0.793	4.33 ± 0.577	4.29 ± 0.463	3.67 ± 0.686	3.26 ± 0.449	1.95 ± 1.146	1.29 ± 0.588
Item 23	3.50 ± 0.707	2.71 ± 0.600	1.41 ± 0.503	3.19 ± 0.849	3.50 ± 0.512	3.17 ± 0.835	4.33 ± 0.577	4.24 ± 0.436	3.67 ± 0.686	3.22 ± 0.518	1.95 ± 1.146	1.24 ± 0.562
Item 24	3.20 ± 0.919	2.54 ± 0.793	1.50 ± 0.512	3.15 ± 0.967	3.50 ± 0.598	3.08 ± 0.793	4.33 ± 0.577	9.10 ± 22.208	3.67 ± 0.686	3.22 ± 0.518	1.90 ± 1.119	1.24 ± 0.562

Abbreviations: F1: primary education/no education; F2: compulsory and noncompulsory secondary education and vocational training; F3: tertiary education/university study.

**Table A6:** Means and SDs of education level obtained on the MDAS and DFS seven days after watching the video.

Scales/Items	Anxiety Disorder			Adjustment Disorder			Mixed Anxiety-Depressive Disorder			No Disorder		
MDAS	F1	F2	F3	F1	F2	F3	F1	F2	F3	F1	F2	F3
Item 1	2.90 ± 1.101	1.89 ± 0.832	1.18 ± 0.395	2.73 ± 0.919	2.68 ± 0.780	2.50 ± 0.674	3.62 ± 0.805	3.81 ± 0.750	3.11 ± 1.023	2.48 ± 0.511	1.60 ± 0.754	1.24 ± 0.437
Item 2	3.00 ± 0.667	2.07 ± 0.663	1.23 ± 0.429	2.65 ± 0.846	2.50 ± 0.740	2.25 ± 0.622	3.71 ± 0.902	3.95 ± 0.865	3.17 ± 1.043	2.52 ± 0.511	1.65 ± 0.745	1.24 ± 0.437
Item 3	3.50 ± 0.707	2.93 ± 0.766	1.95 ± 0.375	2.92 ± 0.796	2.73 ± 0.767	2.42 ± 0.669	4.29 ± 0.956	4.14 ± 0.727	3.22 ± 0.878	2.52 ± 0.511	1.70 ± 0.733	1.41 ± 0.507
Item 4	2.90 ± 0.876	2.18 ± 0.612	1.32 ± 0.477	2.58 ± 1.102	2.36 ± 0.727	2.25 ± 0.622	4.14 ± 0.910	4.19 ± 0.814	3.00 ± 1.085	2.48 ± 0.511	1.55 ± 0.605	1.24 ± 0.437
Item 5	3.30 ± 0.823	2.50 ± 0.694	1.68 ± 0.568	3.00 ± 0.894	3.00 ± 0.690	2.67 ± 0.888	4.33 ± 0.966	4.52 ± 0.814	3.50 ± 0.985	2.61 ± 0.499	1.75 ± 0.786	1.47 ± 0.514
**DFS**												
Item 1	1.80 ± 0.422	1.79 ± 0.568	1.00 ± 0.000	2.42 ± 0.703	2.45 ± 0.671	2.08 ± 0.669	3.00 ± 0.316	3.00 ± 0.548	2.39 ± 0.916	1.96 ± 0.475	1.25 ± 0.444	1.00 ± 0.000
Item 2	1.80 ± 0.422	1.79 ± 0.630	1.00 ± 0.000	2.42 ± 0.809	2.27 ± 0.631	0.17 ± 0.835	3.05 ± 0.590	3.05 ± 0.590	2.28 ± 0.895	1.96 ± 0.367	1.25 ± 0.444	1.00 ± 0.000
Item 3	3.00 ± 0.943	2.32 ± 0.670	1.73 ± 0.550	2.69 ± 0.788	2.59 ± 0.734	2.58 ± 0.669	3.95 ± 0.669	3.71 ± 0.717	3.22 ± 0.878	2.52 ± 0.511	1.85 ± 0.813	1.41 ± 0.507
Item 4	2.90 ± 0.738	2.21 ± 0.738	1.55 ± 0.596	2.77 ± 0.992	2.64 ± 0.658	2.25 ± 0.866	4.19 ± 0.873	3.95 ± 0.740	3.06 ± 0.873	2.57 ± 0.507	1.85 ± 0.813	1.29 ± 0.470
Item 5	2.80 ± 0.632	2.04 ± 0.637	1.55 ± 0.596	2.50 ± 0.860	2.36 ± 0.848	2.08 ± 1.165	4.14 ± 0.727	3.86 ± 0.854	2.89 ± 0.900	2.22 ± 0.518	1.45 ± 0.510	1.18 ± 0.393
Item 6	2.80 ± 0.789	1.93 ± 0.663	1.45 ± 0.596	2.58 ± 0.809	2.27 ± 0.767	1.92 ± 0.900	4.10 ± 0.700	3.95 ± 0.921	3.17 ± 0.924	2.30 ± 0.559	1.50 ± 0.688	1.12 ± 0.332
Item 7	2.90 ± 0.738	2.39 ± 0.786	1.50 ± 0.598	3.00 ± 0.894	2.86 ± 0.710	2.50 ± 0.905	4.62 ± 0.805	4.43 ± 0.926	3.67 ± 1.085	2.70 ± 0.470	2.00 ± 0.918	1.35 ± 0.493
Item 8	2.50 ± 0.972	1.79 ± 0.917	1.09 ± 0.294	2.35 ± 0.797	2.55 ± 0.912	2.08 ± 0.669	3.76 ± 0.436	3.57 ± 0.598	3.17 ± 0.707	2.09 ± 0.515	1.55 ± 0.686	1.18 ± 0.393
Item 9	2.60 ± 1.075	1.93 ± 0.900	1.18 ± 0.395	2.46 ± 0.761	2.59 ± 0.854	2.00 ± 0.739	3.71 ± 0.463	3.52 ± 0.680	3.17 ± 0.618	2.13 ± 0.626	1.55 ± 0.686	1.18 ± 0.393
Item 10	3.10 ± 0.738	2.46 ± 0.693	1.23 ± 0.429	2.42 ± 0.857	2.50 ± 0.859	2.17 ± 0.718	3.81 ± 0.512	3.57 ± 0.746	3.11 ± 0.832	2.04 ± 0.706	1.50 ± 0.688	1.18 ± 0.393
Item 11	4.00 ± 0.943	3.11 ± 0.786	2.09 ± 0.610	3.15 ± 0.784	3.14 ± 0.834	2.92 ± 0.669	4.52 ± 0.602	4.29 ± 0.644	3.72 ± 0.669	2.78 ± 0.671	1.70 ± 0.657	1.47 ± 0.514
Item 12	3.00 ± 0.943	2.57 ± 0.920	1.55 ± 0.858	2.65 ± 0.846	2.86 ± 1.037	2.50 ± 0.674	4.14 ± 0.727	4.24 ± 0.700	3.39 ± 0.916	2.52 ± 0.790	1.90 ± 0.788	1.29 ± 0.470
Item 13	3.50 ± 0.850	2.68 ± 0.819	1.68 ± 0.780	2.65 ± 0.936	2.86 ± 1.125	2.83 ± 1.037	4.29 ± 0.717	4.33 ± 0.577	3.44 ± 1.042	2.35 ± 0.573	1.75 ± 0.716	1.18 ± 0.393
Item 14	2.60 ± 0.966	0.04 ± 0.999	1.14 ± 0.468	2.19 ± 0.981	2.41 ± 0.854	2.33 ± 0.778	3.95 ± 0.590	3.95 ± 0.669	3.28 ± 0.669	2.61 ± 0.783	2.10 ± 0.852	1.41 ± 0.507
Item 15	4.10 ± 0.738	3.54 ± 0.637	2.23 ± 0.752	3.65 ± 0.797	3.77 ± 0.813	3.42 ± 0.793	4.76 ± 0.539	4.86 ± 0.359	4.11 ± 0.900	3.22 ± 0.600	2.50 ± 0.827	1.88 ± 0.485
Item 16	2.40 ± 1.075	1.64 ± 0.870	1.05 ± 0.213	2.08 ± 0.977	2.23 ± 0.752	2.08 ± 0.669	3.81 ± 0.402	3.71 ± 0.644	3.33 ± 0.840	2.39 ± 0.722	1.80 ± 0.696	1.41 ± 0.507
Item 17	2.90 ± 0.876	1.93 ± 0.858	1.23 ± 0.429	2.31 ± 1.087	2.14 ± 0.774	2.42 ± 0.793	4.10 ± 0.768	3.95 ± 0.669	3.50 ± 0.786	2.48 ± 0.730	2.05 ± 0.759	1.47 ± 0.624
Item 18	4.40 ± 0.699	3.43 ± 0.790	2.41 ± 0.590	3.62 ± 0.852	3.55 ± 1.101	3.33 ± 0.985	4.176 ± 0.539	4.76 ± 0.539	4.11 ± 1.023	2.91 ± 0.596	2.35 ± 0.587	2.00 ± 0.500
Item 19	2.90 ± 0.994	2.00 ± 0.981	1.36 ± 0.492	2.00 ± 1.058	2.18 ± 0.795	2.17 ± 0.718	3.62 ± 0.590	3.86 ± 0.727	3.11 ± 1.231	1.65 ± 0.885	1.40 ± 0.681	1.06 ± 0.243
Item 20	3.80 ± 0.422	3.39 ± 0.737	2.23 ± 0.612	3.42 ± 0.987	3.36 ± 0.727	3.08 ± 0.900	4.81 ± 0.512	4.86 ± 0.359	4.17 ± 0.985	3.35 ± 0.487	2.45 ± 0.759	1.94 ± 0.243
Item 21	3.80 ± 0.422	3.39 ± 0.737	2.23 ± 0.612	3.50 ± 0.949	0.27 ± 0.767	3.17 ± 0.835	4.81 ± 0.512	4.86 ± 0.359	4.28 ± 0.958	3.35 ± 0.487	2.40 ± 0.821	2.00 ± 0.354
Item 22	3.40 ± 0.699	2.32 ± 0.819	1.14 ± 0.503	3.04 ± 0.871	3.32 ± 0.646	2.92 ± 0.900	4.19 ± 0.602	4.29 ± 0.561	3.61 ± 0.778	3.26 ± 0.449	1.90 ± 1.119	1.24 ± 0.562
Item 23	3.40 ± 0.699	2.32 ± 0.819	1.36 ± 0.492	3.04 ± 0.871	3.18 ± 0.664	3.00 ± 0.953	4.19 ± 0.602	4.29 ± 0.561	3.61 ± 0.778	3.22 ± 0.518	1.85 ± 1.089	1.24 ± 0.562
Item 24	3.20 ± 0.919	2.29 ± 0.763	1.32 ± 0.477	3.00 ± 0.894	3.18 ± 0.733	2.92 ± 0.900	4.14 ± 0.573	8.95 ± 21.327	3.61 ± 0.778	3.17 ± 0.576	1.85 ± 1.089	1.24 ± 0.562

Abbreviations: F1: primary education/no education; F2: compulsory and noncompulsory secondary education and vocational training; F3: tertiary education/university study.

**Table A7:** Reliability of scales construct validity and factor extraction for the three video viewings.

Scales/Items	Viewing Times	Reliability of Scales (Alpha Cronbach)	Construct Validity (KMO Index)	Factor Extraction (Varimax Rotation) and Aggregated Items	Accumulated Variance
Modified dental anxiety scale Item 1: How would you feel if you had to go to the dentist for treatment tomorrow? Item 2: How would you feel sitting in the waiting room (waiting for your dental treatment)? Item 3: How would you feel if you were about to have a tooth drilled? Item 4: How would you feel about removing plaque from your teeth and then having them polished? Item 5: How would you feel if you were about to receive an injection of anesthetic in your gum above one of the upper teeth in the back of your mouth?	Seven days before After the video Seven days after	0.939 0.954 0.967	0.962 0.868 0.870	Group into 1 factor: UNIDIMENSIONAL Group into 1 factor: UNIDIMENSIONAL Group into 1 factor: UNIDIMENSIONAL	80.578% 85.284% 88.406%
Dental fear survey Item 1: Has fear ever caused you to (made you) postpone a dental appointment? Item 2: Has a fear of a dental appointment ever caused you to cancel or not attend an appointment? Item 3: When I am being treated at the dentist: My muscles are tense…. Item 4: I breathe faster… Item 5: I perspire… I sweat…. Item 6: I feel nauseous and sick to my stomach… Item 7: My heart beats faster… Item 8: Asking for a dentist’s appointment. Item 9: Going to the dental clinic. Item 10: Sitting in the waiting room. Item 11: Sitting in the dentist’s chair. Item 12: Smelling the smell of the dentist’s office. Item 13: Seeing the dentist come in. Item 14: Seeing the needle with anesthetic. Item 15: Feeling the needle with an anesthetic. Item 16: Seeing the drill (handpiece or turbine). Item 17: Hearing the drill. Item 18: Feeling the vibration of the drill. Item 19: Having clean teeth. Item 20: On considering all these things, what are you afraid of in dental treatment? Item 21: How much fear does dental treatment generally cause you? Item 22: How afraid was your mother? Item 23: How afraid was your father? Item 24: How afraid were your siblings?	Seven days before After the video Seven days after	0.984 0.890 0.926	0.841 0.964 0.957	Group into two factors: Factor 1: Items 8–19. Factor 2: Item 1–7, 20–24. Group into two factors: Factor 1: Items 3–19, 21, and 22. Factor 2: Items 1, 2, and 5. Eliminated items: 20 and 24. Group into two factors: Factor 1: Items 1–23. Factor 2: Item 24.	79.281% 71.523% 79.137%
